# Humanitarian led community-based surveillance: case study in Ekondo-titi, Cameroon

**DOI:** 10.1186/s13031-021-00354-9

**Published:** 2021-03-26

**Authors:** Alain Metuge, Lundi-Anne Omam, Elizabeth Jarman, Esther Omam Njomo

**Affiliations:** 1 Reach Out N.G.O , https://www.reachoutcameroon.org; 2 Department of Public Health and Primary Care, University of Cambridge, Cambridge, UK ,

**Keywords:** Community based surveillance, Conflict, Cameroon, Community health workers

## Abstract

**Background:**

Community-based surveillance (CBS) has been used successfully in many situations to strengthen existing health systems as well as in humanitarian crises. The Anglophone crisis of Northwest Southwest Cameroon, led to burning of villages, targeting of health personnel and destruction of health facilities which, in combination with distrust for the government services led to a collapse of surveillance for outbreak prone diseases.

**Methods:**

We evaluated the ability of the CBS system to identify suspected cases of outbreak prone diseases (OPD) as compared to the facility-based surveillance, evaluated the timeliness of the CBS system in identifying an OPD, reporting of OPD to District Health Service (DHS) and timeliness in outbreak response. The paper also assessed the collaboration with the DHS and contribution of the CBS system with regards to strengthening the overall surveillance of the health district and also determine the interventions undertaken to contain suspected/confirmed outbreaks.

**Results:**

In total 9 alerts of suspected OPDs were generated by the CBS system as compared to 0 by the DHS, with 8 investigated, 5 responses and 3 confirmed outbreaks. Average time from first symptoms to alert generation by the CBS system was 7.3 days. Average time lag from alert generation from the CBS to the DHS was 0.3 days which was essentially within 24 h. There was extensive and synergistic collaboration with the DHS.

**Discussion:**

CBS generated a higher number of alerts than traditional outbreak reported used in the region, and had timely investigations and if appropriate, responses. Careful selection of CHWs with strong community engagement led to the success of the project, and the use of the mobile health team in situ allowed for rapid responses to potential outbreaks, as well as for feedback to CHWs and communities. CBS was also well utilized for identification of other events, such as displacement and malnutrition.

**Conclusion:**

In conflict settings, CBS can help in outbreak identification as well as other events, and a mobile health team is crucial to the success of the CBS due to the ability to rapidly response to generated alerts. The mobile health team provided timely investigation of 8 of 9 alerts generated. Collaboration with existing DHS structures is important for systems strengthening in such settings.

## Background

Community-based surveillance (CBS) is defined as the systematic detection and reporting of events of public health significance by community members within a community [1]. CBS has been used in both; non crisis settings for public health emergencies and crisis situations including conflict and natural disasters [2–4]. CBS has been used in eradication programs including guinea worm and polio, as well as data for maternal mortality, mortality from infectious diseases and malnutrition [2, 5–7]. CBS systems have been reported in emergencies around the world, contributing to improving emergency programming in communities. The increasing use of CBS in conflict-affected areas is reshaping humanitarian response in ways by timely reporting of specific information to those who offer assistance [3].

CBS was developed to overcome logistical difficulties of travel and communication, which are common in low-and-middle income countries which constrain the conventional surveillance system reliant on epidemiologists visiting sites to discover and investigate cases, particularly in rural areas [5]. CBS can be cost effective as community members are often willing to report for little financial compensation leading to low costs for information gathering. CBS is recommended by the World Health Organization (WHO) for use in displacement camps, complex emergencies and outbreaks [3], and can function in the pre-epidemic period, during epidemics and post epidemic period [8]. Despite the benefits of CBS, there are many challenges associated with it, as identified by *Ratnayake* et al. These included but are not limited to; decreasing acceptability from communities resulting from the weariness of constantly being asked about loss of life, weekly home visits with no direct benefits to households, CHWs experiencing tiredness and threats from community members towards CBS workers [3]. In addition, enumeration of populations is challenging in some settings, especially in areas where due to protection incidents, certain populations do not want to be found [9].

Conflict and other crisis affected populations face a myriad of humanitarian problems and needs including population displacement, separation of families, food insecurity, absence of health services and increase vulnerability to outbreaks. The destruction of health facilities and the displacement or death of health facility staff members leads to a collapse of the health system, including immunization services and disease surveillance.

There is little evidence available on the use of CBS in conflict affected communities of Cameroon since the armed crisis dubbed “the Anglophone Crisis” started in the Northwest Southwest (NWSW) regions in 2016. Though a couple of humanitarian organisation might be using it to activate early warnings of outbreaks. It is estimated that more than 200 villages have been destroyed or abandoned due to the conflict. The Office for the Coordination of Humanitarian Affairs (OCHA) estimated in December 2019, 679,000 people were internally displaced in NWSW Cameroon [10]. The insecurity, displacement of health professionals and targeted attacks on health facilities has led to the closure of 37% of health centres in the NWSW, a situation which is replicated in Ekondo-Titi health district [11]. Prior to 2016, the district had 13 health facilities and one hospital serving a population of 56,503 inhabitants. By March 2019, the number of functional health facilities had dropped to 4 [12]. Surveillance, which relied upon health facility reporting, deteriorated in the health district as it did across NWSW Cameroon. In 2019, 9 health districts in the Southwest did not report a suspected case on a measles, neonatal tetanus, yellow fever or AFP [13], and within Ekondo-Titi health district no suspected cases were identified in 2015, 2016, 2017 and 2018. This is most likely due to reduced surveillance rather that true lack of outbreaks as there were also no false reports. In addition, community acceptance of government facilities decreased, and it became increasing difficult for government workers to continue to live or work in these areas, which led to a drop-in surveillance due to lack of presence. Prior to the Anglophone Crisis, disease surveillance in the health district followed the national Integrated Disease Surveillance System. It involved the district collecting surveillance data from all health facilities within the district based on patients seen at these facilities which is then sent to the Regional Delegation of Public Health for compilation and forwarding to the National Surveillance Unit. In the absence of health facility workers and functional health facilities, the surveillance system for most districts in NWSW Cameroon collapsed. The attacks on and burning of villages caused people to flee, led to family separation with reports of unaccompanied and separated children [10].

In the light of these happenings Reach Out NGO, with support from UNICEF implemented a one-year project called “Rapid Response Mechanism (RRM+) for timely emergency response to displaced communities in hard-to-reach areas” within Ekondo-titi Health district. In a bid to improve the identification and notification of outbreaks and occurrence of diseases under the National Surveillance Program, CBS was included in the project activities. This paper would address some of the challenges identified for CBS at the WHO meeting in 2018, namely accessing the most hard-to-reach places, and challenges around enumeration of populations [1]. This paper presents an evaluation of the CBS system used in the RRM+ project in improving disease surveillance and health outcomes and strengthening the existing district surveillance in conflict affected communities. Specifically, this paper seeks to evaluate the ability of the CBS system to identify suspected cases of outbreak prone diseases (OPD) as compared to the District Health Service (DHS) facility-based surveillance, evaluate the timeliness of the CBS system in identifying an OPD, and timeliness in outbreak response were appropriate. The paper will also assess the collaboration with the DHS and contribution of the CBS system with regards to strengthening the overall surveillance of the health district and also determine the interventions undertaken to contain suspected/confirmed outbreaks. We also describe the CBS system approach along with the challenges and results which could be replicated for scale up.

## Methods

The CBS system was set up as part of the RRM+ project in Ekondo-titi and it was operational from July 2019 to April 2020, although the RRM+ project itself started in April 2019. The evaluation was carried out after the end of the project and period was from May 2020 to June 2020.

We explain below how we assessed each objective in this paper;

We evaluated the ability of the CBS system to pick up suspected cases of OPDs as compared to the DHS facility-based surveillance. This was done first by checking the district’s data base for disease outbreaks reported by the district from January 2016 to July 2019 in order to set the baseline average disease outbreak reports from Ekondo-titi. We checked the DHS reports on any suspected OPD cases notified in the period July 2019 to April 2020 and RRM+ project reports. Suspected cases of OPD first reported to the DHS by the CBS system mobile health team (MHT) were credited to the CBS system and those first reported from health facilities to the district were credited to the DHS facility-based surveillance.

We evaluated the timeliness of the CBS system. We assessed how soon the CBS system picked up the suspected cases. This was assessed by evaluating outbreak notification, patient history, and field mission reports from the MHT. The patient history contained information on the date the patient or parent reported the first symptoms. For all the reported outbreaks the MHT was able to record the date first symptoms started and where these dates of first symptom could not be identified, the date the parents first noticed physical changes like rashes was considered. From these documents, date of first symptom were extracted and placed against time of identification by CHW who reported the suspected outbreak. We verified response time after notification. This was extracted from MHT notification and outbreak investigation mission reports. Verification response time was the time lag between when the suspected outbreak was reported to the MHT to when the report was investigated and samples collected.

We evaluated the collaboration with the DHS and assess the contribution of the CBS system with regards to strengthening the overall surveillance of the health district. Here we looked at all outbreak alerts picked up by the RRM+ CBS system and checked with the DHS if they were alerted by the MHT and how soon. We also assessed the role of DHS in responding to each report of a suspected or confirmed outbreak.

Finally, we evaluated the interventions undertaken to contain suspected/confirmed outbreaks. This was done by looking at the actions taken by the MHT to contain suspected or confirmed outbreaks by evaluating the field mission reports, weekly, and monthly reports.

### Study site

Ekondo-titi health district has a population of 56,503 inhabitants and nine health areas. Three of these health areas Illor, Bamusso and Bekumu are primarily maritime areas, while the other 6 health areas, which include Ekondo titi, Lobe, Bekora, Kumbe Balue, Bafaka Balue, and Bissoro health areas are mainland communities. The RRM+ was implemented in all the above health areas with the exception of Bamusso and Bissoro health areas because these were not affected by the crisis and had functional health facilities.

The goal of the RRM+ project in Ekondo-titi health district was to reduce suffering and improve the coping mechanisms of the affected population. Though the main benefit package was health delivery, the needs of populations in humanitarian settings had to be tackled from a holistic approach. Thus, the project also included nutrition, child protection, and water, sanitation and hygiene components. These had to be implemented through the use of a MHT made up of a medical doctor, 2 nurses, and a protection officer. The MHT was the first-line response team for any health-related event within the health district. For this to be effective the team needed to set up a CBS system in the health district which could effectively monitor and report health events and cases. The list of diseases that were included for monitoring in the CBS were OPD under surveillance in Cameroon and endemic diseases and other events. These are document in Table 1 and were selected based of high mortality rates, endemicity and epidemic potential.

**Table 1:** Outbreak prone diseases under surveillance in Cameroon and endemic diseases and health events monitored in the project

Health event / disease	Case Definition	Rational for including
Outbreak prone diseases		
Acute Flaccid Paralysis	Any child with a sudden onset of acute paralytic disease.	Disease for eradication
Measles	Any person with elevated body temperature and widespread rashes on the face and the body	Epidemic-prone due to poor vaccination coverage
Suspected case of Cholera	Any person aged 5 years or more who has lots of watery diarrhea	Epidemic prone and potential high mortality
Buruli ulcer/Tropical ulcer	Any person with an ulcer that fails to heal after 2 weeks	Endemic disease in the district with severe deforming complications
Meningitis	Any person with fever and a stiff neck.	Epidemic prone and potential high mortality
Endemic diseases and other events		
SAM	Any child 6-59 months old with a MUAC less than 115 mm	Endemic and high associated mortality
Displacement	Sudden movement of at least 50 or more people or 10 house holds	Need to track populations in urgent need of support due to rapid displacements, loss of shelter etc
Uncomplicated malaria	Any individual with a fever within 24 h	Endemic and high associated mortality
ARI in children under 5 years old	Fever plus cough or catarrh or difficulty breathing	Endemic and high associated mortality
Acute watery diarrhea	3 or more watery stools within 24 h	Endemic and high associated mortality
Neonatal tetanus	Any neonate between 3 to 28 days old who cannot suck normally and becomes stiff	High associated mortality and increased risk due to reduced numbers of births in facilities

For the RRM+, community health workers (CHWs) were the main field agents at the community level as they were community members who had been internally displaced with the affected populations. The majority of these CHWs (34 of 40) had previously worked in the role as CHWs of the DHS, but 6 were newly recruited in areas where the district had not assigned any trained CHW. These CHWs served as CBS focal persons, following training, and reported to the MHT.

The MHT verified all reports, collected samples where applicable (including stool for cholera or acute flaccid paralysis, whole blood for measles, or swabs for monkeypox or Buruli ulcer), led public health interventions in response to confirmed disease outbreaks, provided feedback to the CHWs and the community. The MHT also informed relevant stakeholders including the DHS and the Regional Delegation of Public Health.

### Design of the system

#### Surveillance of outbreak prone diseases, endemic disease and other events

CBS was intended to complement and bridge the gap in surveillance in areas that routine surveillance was partly or non-functional in the notification of OPD. Surveillance in our project also included monitoring and notification of endemic health issues like malaria, acute respiratory infections (ARI), acute diarrhea, chronic ulcers, and conflict related events like security incidents and population displacements which influence the health of affected populations. The OPD selected were chosen based on their likelihood of occurrence brought about by the conflict and also taking into consideration the associated mortality. For endemic health disease monitoring, it should be noted that the diseases monitored are all disease associated with the greatest under five mortality in Cameroon and conflict settings. Buruli ulcer is endemic in Ekondo-titi and in the context of conflict there is no active surveillance and this could lead to deformities amongst patients who have no access to healthcare. Population displacements were monitored because of the interconnectedness of health, nutritional, water, sanitation, and shelter need and displacement. It was critical to gather this information to prepare for health response in emergency displacements. CHWs were expected to report suspected cases of OPD and other public health events, with OPDs reported immediately, and other endemic or events reported less frequently as OPDs need accurate and timely surveillance and response. For this CBS system a single suspected case of any OPD was sufficient to generate an alert. However, if multiple suspected cases of the same OPD occurred in a single community and reported within the same period, it only counted as one generated alert. The definition of an outbreak is disease specific and is in accordance with the national guidelines. For measle it is four confirmed cases within the same health district within the same time period. For cholera, a single culture confirmed case constitutes an outbreak, similarly for monkeypox.

Suspected cases of measles, cholera, meningitis, neonatal tetanus, AFP were reported immediately through a phone call or text message on the CHWs personal phone, whereas other diseases (diarrhea, malaria and ARI) were reported weekly. The case-fatality rates of the aforementioned diseases necessitated a quick response to control their spread. There was no electronic data collection tool available for CHWS for reporting OPD, due to the extremely poor quality of signal within the district. In some cases, in the absence of network, CHWs walked, sometimes as far as 20 km, to the Reach Out field office to report a case. On receipt of this information by the MHT, and completion of verification, national reporting tools were completed and submitted to the DHS to strengthen local reporting. All data used in the evaluation was collected from RRM+ project outbreak alert reports, field mission reports, weekly and monthly project reports, and DHS reports.

Surveillance on malnutrition was carried out in all selected health areas. CHWs screened every child 6 to 59 months of age during home visits and or any consultation session. They reported number of children screened weekly, number of children 6 to 59 months of age with severe acute malnutrition (SAM), 6 to 59 months with moderate acute malnutrition (MAM). All children with SAM without complication were managed in the community by CHWs under the supervision of the MHT which visited at least once a month to monitor all SAM cases and also provide nutritional counselling to parents and caretakers of children diagnosed with SAM and MAM. All components of the CBS system started simultaneously.

#### Selection of CHWs

The selection of CHWs was done with the assistance of the DHS and the local leaders of displaced communities. The health district provided a list of CHWs per village as per before the conflict. The MHT then visited each community where local stakeholder engagement meetings were held to confirm the appropriateness of the selected CHW, based on presence in the community and local acceptance. Communities who lacked a CHW due to displacement or lack of local health personnel, identified a member of the community who had good local acceptance for the role. A total of 40 CHWs from 20 communities were identified to be trained in the project. The rational for choosing 40 CHWs was that this was aiming for 1 CHW to cover 500 persons. However, due to poor access to data on displacement as no humanitarians had previously worked in the area, this ended up being quite inaccurate, with some CHWs covering 3 times as many beneficiaries. CHWs had previously been trained in iCCM (integrated community case management) by the National Malaria Program under the Global Fund to fight AIDS, tuberculosis and malaria subvention. However, these CHWs were mostly managing uncomplicated malaria, diarrhea and ARI, and conducting health education on HIV, family planning, nutrition and immunization.

### Training of CHWs

In addition to their iCCM training packages as CHWs, the CHWs were also trained on CBS. Their training was developed from the WHO Community-Based Surveillance Training Manual [14] and they were trained for 4 days [8]. This training included case definitions for malaria, acute diarrhea, bloody diarrhea, cholera, respiratory tract infection, moderate acute malnutrition, severe acute malnutrition, yellow fever, neonatal tetanus, chicken pox, chronic ulcer, measles, poliomyelitis, and meningitis. In addition, they were trained on humanitarian principles, security, the prevention of sexual exploitation and abuse, and accountability for affected populations.

CHWs were trained to use a mid-upper arm circumference (MUAC) measurement to identify SAM and MAM cases. There was no local treatment centre or height or length boards available.

#### Data collection

Surveillance was done by CHWs who collected data primarily through door to door visits in the first 6 to 8 weeks. However, with time it became a combination of home visits and direct reporting of cases by community members to the CHWs. Diseases under surveillance were selected by the project team based on most common epidemic prone diseases locally, and are presented along with the case definitions on Table 1.

Weekly reporting tools were designed to capture the surveillance data for diseases that didn’t require to be reported immediately, such as malaria, diarrhoea and ARI. Each CHW submitted their weekly report at the field office in Ekondo-titi to the MHT. It became apparent that, some CHWs could not travel from their communities to the field office every week because of extremely poor roads, expensive use of boats and insecurity of the roads due to frequent gun battles between the military and non-state armed groups (NSAG). This was the case of communities like Bekumu, Bafaka, Kumbe Balua, Ekue Balue, Illor Balundo and Bongogo 2. CHWs were also trained on how to submit their weekly reports using their personal phones through text messages, which were sent to the MHT. No standardised format for reporting using mobile phones was developed. Data received from CHWs were logged into and analysed using Microsoft Excel on a weekly and monthly base by the MHT.

#### Supervision of CHWs

CHWs were supervised by the MHT, who were also responsible for field verifications, collection of samples and led the public health interventions related to the reported disease outbreaks or other public health events in collaboration with the DHS and the Regional Delegation of Public Health. The MHT carried out monthly field supervision visits to assess field work, build capacity and review cases.

##### Ethical consideration

This evaluation was part of a non-research public health response project, framed as a public health evaluation to improve practices and thus was not reviewed by an ethics review board. More so, the interventions under this project were done under normal public health practices and all data as collected as part of routine data collection and was anonymized for the evaluation.

## Results

From January 2016 to June 2019 the DHS surveillance system generated no alert for an OPD. In total, 8 alerts of OPD were generated by the RRM+ CBS system as compared to 0 by the DHS during the evaluation period which was from July 2019 to April 2020 (Table 2). Of these 8 alerts, 7 were investigated by the MHT, and 5 responses occurred to 3 confirmed outbreaks (Table 4). Table 2 shows the OPD alerts generated and also the endemic diseases monitored by the CBS system. The CBS helped identify and manage 6482 cases of uncomplicated malaria, 2310 ARI, 1882 cases of acute diarrhea, 13,177 screenings of children aged 6–59 months were conducted, identifying 43 cases of SAM.

**Table 2:** Number of reports, alerts generated, and outbreaks recorded

	Number of cases reported by MHT	Number of alerts generated by MHT	Number of outbreaks reported by MHT	Number of cases reported by DHS	Number of alerts reported by DHS	Number of outbreaks reporting by DHS
Outbreak prone diseases						
AFP	0	0	NA	0	0	0
Measles	7	3	1	0	0	0
Suspected case of Cholera	9	2	1	0	0	0
Buruli ulcer/ tropical ulcer	21	1	1	0	0	0
Meningitis	4	1	0	0	0	0
Monkeypox	1	1	1	0	0	0
Endemic diseases						
SAM	43	NA	NA	NA	NA	NA
Uncomplication malaria	6482	NA	NA	NA	NA	NA
ARI in children < 5 years	2310	NA	NA	NA	NA	NA
Acute watery diarrhea	1882	NA	NA	NA	NA	NA
Neonatal tetanus	0	NA	NA	NA	NA	NA

The CBS reported suspected cases for 9 diseases (Table 2) and the CBS system had an alert generation time which ranged from a minimum of 2 days to a maximum of 18 days. The average delay from the day of first symptom to alert generation by the CBS system was 7.3 days (Table 3). This broad average reporting time can be explained by some outliers. The case definition for chronic ulcers required that it last more than two weeks to generate an alert leading to a 16 lag-time from first symptoms. Also, patient zero for the measles alert lived in a community not covered by the project and was only seen upon visiting Loe were the disease spread, his time lag was 18 days from first symptoms. Such large variations greatly influenced the average reporting time (Table 3). Response to investigate OPD alerts by the MHT ranged from 1 days to a maximum of 2 days from the day of alert generation by the CBS system (Table 3).

**Table 3:** Suspected outbreak prone diseases reported by CHWs under the RRM+ CBS system in Ekondo-titi, time to reporting and time to investigation

Community	Suspected OPD	Cases reported	Cases investigated	Samples collected	Positive case	Symptom onset	Date alert generated	Date DHS informed	Date MHT Investigated	Lag time symptom to alert	Lag time alert to response	Time lag for information of DHS
										**(Days)**		
Loe	Measles	4	4	3	3	29/7/19	2/8/19	2/8/19	3/8/19	4	1	0
Mokono	Measles	1	1	1	1	15/7/19	2/8/19	2/8/19	3/8/19	18	1	0
Ndoo	Measles	1	1	1	0	1/8/19	4/8/19	4/8/19	6/8/19	3	2	0
Funge	Chronic ulcers	21	21	21	19 tropical ulcers	20/8/19	6/9/19	6/9/19	8/9/19	16	2	0
					2 Burulli ulcers	20/8/19	6/9/19	6/9/19	8/9/19		2	0
Illor	Measles	1	1	1	1 Monkey pox	7/9/19	14/9/19	17/9/19	15/9/19	7	1	3
Bafaka	Meningitis	4	2	1	1	24/3/20	31/3/20	31/3/20	2/4/20	7	2	0
Bongongo 2	Measles	1	0^a^	0	0	4/8/19	6/8/19	6/8/19	–	2	–	0
Godgift	Cholera	2	5	3	1	25/1/20	29/1/20	29/1/20	30/1/20	4	1	0
Bamusso	Cholera	7	7	3	0	12/1/20	17/1/20	17/1/20	18/1/20	5	1	0

^a^Patient relocated

Feedback to communities was done through CHWs in cases where there was no field response after the investigation and by the MHT during field response for confirmed outbreaks.

### The MHT collaboration with the DHS

Local health administrators were unused to working with humanitarian actors and initial relationships were challenging, especially with regards to outbreak reporting. As the districts reporting had been mostly silent, the sudden influx of diseases identified when humanitarian actors began working was viewed negatively. This required regular and extensive engagement, updating the local district authorities on all suspected cases as we received them and involved them in the response by updating them regularly of our activities. Table 3 show that alerts generated by the CBS system were shared with the district within 24 h of the alert generation. This was true for all cases with the exception of the suspected measles outbreak alert case from Illor which after initial phone conversation with the CHW, the MHT suspected a case of chicken pox based on the phone description of the CHW. It was only after the laboratory confirmed the test results as being positive for monkeypox that the MHT alerted the DHS 3 days later. This explains the delay seen on the Table 4 for that single case. Average time lag from alert generation to information of the DHS was 0.3 days which was essentially within 24 h.

**Table 4:** Role of RRM+ CBS and DHS in OPD and endemic diseases identified in Ekondo Titi health district, investigation and response

Community	Suspected disease	Cases reported	Investigations	Response per suspected outbreak
Loe	Measles	4		Whole blood samples collected and sent to reference laboratory Community sensitization, mobilization and ring vaccination of 317 children with the measles, mumps, rubella vaccine
Mokono Beach	Measles	1		
Ndoo	Measles	1		
Funge	Chronic ulcers	21	Gram stain of ulcer swaps	Increase community sensitization on Buruli ulcer and tropical ulcer. The 2 Buruli ulcer cases were referred to the treatment center at the district hospital. The 19 tropical ulcer cases were managed in the community by a trained local nurse.
Illor	Monkeypox	1	Whole blood and lesion swaps Polymerase chain reaction of both samples	Patient was isolated and preventive measures put in place to prevent the spread of the disease. Patient was managed symptomatically for a month until all lesions and symptoms resoled. Contact tracing was done and 32 people monitored for 21 days.
Bafaka Balue	Meningitis	4	Cerebrospinal fluid (CSF) collected CSF examination, gram stain	Samples were only positive for non-epidemic meningitis. Patients where referred to the district hospital where they were managed appropriately
Bongongo 2	Measles	1	NA patient had moved out of the district	NA
Godgift	Cholera	2	Rapid diagnostic tool test	No Cary Blair transport medium was available. Community education was carried out and the people were provided sufficient water purification tablets to last each household for at least a month.
Bamusso	Cholera	7		

There was extensive collaboration with the DHS. While the CBS system generated all the alerts, the district was informed within 24 h in all cases and response was collaborative between the MHT and the DHS. MHT conducted all field investigations with supplies provided by the DHS. Samples brought from the field were transported to the reference labs by the DHS and results from lab was brought back to the MHT by the DHS. While the MHT designed and executed all field responses, some supplies required for effective and quality response like vaccines, vaccines carriers and syringes were provided by the DHS. Table 3 show that for all OPD alerts the MHT conducted a field investigation mission, collected samples and carried out a community response for all confirmed and all suspected outbreaks. This additional usefulness of the system is because this CBS wasn’t designed to just identify the cases for the purpose of pure surveillance. This CBS was to allow early identification, reporting, investigation and response. Given that health centers in the districts weren’t functional and the DHS doesn’t normally have any allocated resources for an investigation and response, the MHT had to play the critical role of being ready to move as soon as they received an alert, investigate the alert, collect samples which were then handed to the DHS to be sent to the reference laboratory. When results were available, the MHT in collaboration with the DHS planned an appropriate response.

### Results of interventions undertaken to contain suspected/confirmed outbreaks

#### Measles outbreak

The MHT upon reception of the alert informed the DHS on the same day. All other CHWs were alerted to be more vigilant in their search for suspected measles cases. The MHT visited Loe the subsequent day for investigation and sample collection in collaboration with the DHS. The DHS provided the cold box and icepacks for protection of the samples. The children were examined and samples were collected by the MHT who also prepared the CHWs and community of the likelihood of an emergency measles vaccination campaign should the samples comeback positive for measles. The MHT also carried out a brief community sensitisation on measles. Samples were sent to reference lab in collaboration with the DHS. After 3 days the MHT were informed by the DHS that 4 of the 5 samples were positive for measles, meeting the national criteria for a measles outbreak. The MHT mobilised CHWs in communities within the affected health areas and other neighbouring communities informing them on the outbreak and the need for an emergency ring vaccination campaign. With vaccines provided by the DHS, the MHT conducted a ‘ring vaccination’ intervention for children in Loe and surrounding communities prior to the laboratory confirmation of the measles cases. A total of 317 children aged 6 months to 14 years were vaccinated during this ring vaccination intervention against measle, mumps and rubella, and administered with vitamin A. No further cases of measles were identified after this ring vaccination.

After the measles outbreak was confirmed, a vaccination campaign for the entire district was only organised 4 months later.

#### Monkeypox outbreak

A CHW raised an alert for a suspected case of measles to the MHT. The MHT on discussion diagnosed a likely severe case of chicken pox and the CHW was asked to refer the case to the Ekondo-titi district hospital. The patient, a 14-year old boy presented after 2 days and was managed by the hospital. One day after arrival at the hospital, the MHT went in to see the patient and after reviewing the patient’s history and physically examining the characteristic lesions, the MHT made a tentative diagnosis of a suspected monkeypox case. The MHT immediately alerted the hospital administration of their findings. With assistance from the MHT, the patient was immediately placed in isolation with limited contact from both family and hospital staffs. A disinfection unit was set outside the isolation room for those who had contact with the patient to disinfect their hands as they leave the patients room. The MHT then informed the DHS, collected blood samples and swabs from the lesion for analysis. Samples were handed over to the DHS for transportation to the reference laboratory at central level. The MHT then conducted contact tracing of all individuals who had contact with the patient. A total of 32 people were identified including hospital staffs, a member of the MHT, 2 CHWs, family members and neighbours, who were asked to inform the MHT or report to the district hospital if they developed any rashes within 21 days. 4 days later the DHS reported the virology results had confirmed the case to be a monkeypox case. The patient was managed in isolation for another 2 weeks until all lesions had healed completely. Those on the contact list were called weekly for 3 weeks to follow up if anyone had developed symptoms of monkeypox and no other cases were identified in 21 days.

#### Buruli ulcer/ tropical ulcer outbreak

An outbreak of 21 new cases of chronic leg ulcers amongst children 5–15 years were reported by a CHW from Funge. The MHT immediately informed the DHS, who provided the MHT with swab sticks, a cold box and ice. On arrival the MHT examined all 21 patients and collected the samples. Based on the history and characteristics of the ulcers, the mobile team diagnosed tropical ulcers for all 21 cases. The samples were handed over to the district lab where only 2 were positive for Buruli ulcer. The Buruli ulcer cases were referred to the treatment center at the district hospital. The tropical ulcer cases, whose diagnoses had been made base on the classical presentation and exclusion of Buruli ulcer, were managed at community level by a community nurse who was trained by the MHT on regular and proper wound cleaning, in combination with single dose Azithromycin for all 19 children. Community education and sensitization on Buruli and tropical ulcers were intensified with focus on the causes, means of transmission and prevention.

#### Suspected cholera outbreaks

For the 2 suspected cholera outbreaks, the DHS in collaboration with the MHT responded promptly to investigate the reports of at least 2 deaths suspected to be cholera related before arrival. The MHT carried out community assessment in relation to the suspected outbreaks. Patients were taken to the nearest health facilities which though under equipped served as treatment centers, where rapid diagnostic tests (RDTs) for cholera were carried out and results were negative. No Cary Blair transport medium was available. Community education was carried out and the people were provided sufficient water purification tablets to last each household for at least a month.

#### Suspected meningitis outbreak

As for the suspected meningitis outbreak, the MHT investigated and successfully collected a CSF sample from one patient. A gram stain was done which revealed the presence of gram-positive cocci and gram-positive diplococci. Though the sample was positive for meningitis it wasn’t the epidemic type of meningitis. Patients were referred to the Ekondo-titi district hospital and managed appropriately.

### Displacements and security incidents

Throughout the RRM+ project, all communities covered by the project kept shifting states from being internally displaced persons (IDPs) living in bushes or in other host communities, to returnees and letter becoming host communities themselves. Security alerts and population displacements were captured through the CHWs weekly and monthly reports and the MHT’s communication on security events in their immediate and neighboring communities. A total of 15 security events and 16 reported incidents of sudden population displacements were recorded amongst 10 communities. All reported security events where either gun battle encounters between the military and the NSAG, forceful retention, and/or violent harassments. Adding to the visibility materials like badges and project jackets which were provided for all CHWs, they had been trained on security and how to best communicate and or react during a security incident. However, few incidents of harassments were recorded from both military and the NSAGs. One CHW had their house broken into and some of the drugs stolen. Insecurity, poor telecommunication signals, and extremely bad roads where the most common challenges reported by CHWs and the MHT. The safety of CHWs in this project was treated we utmost importance due to frequent security incidences in Ekondo-titi. First, all CHWs were trained on safety and security and humanitarian principles to enable them have foresight on what to do when faced with a security threat. Access negotiations and mediations were done with both the government and community leaders on behalf of both the CHWs and mobile health team. CHWs were told to always have their visibility jackets on for easy identification during targeted incidences.

## Discussion

Results from the evaluation of the CBS system proved useful to both the humanitarian response and strengthening the district surveillance and health event response. Given that no case for an OPD was reported and therefore no alert generated by the DHS over a three-year period from when the crisis began in 2016 to the time the CBS system was created in June 2019, and that the CBS system generated 9 alerts as compared to no alert reported by DHS during the period under evaluation. All but one of the 9 generated alerts were investigated and all investigated alerts were responded to by the MHT. CBS filled a gap in surveillance for early detection of suspected outbreaks in remote and insecure areas, given that the DHS surveillance did not detect any alerts in the prior period. Our results corroborate with findings from *Kisanga* et al [12] whose evaluation of the CBS system in South Sudan, proved to be effective in increasing the number of reported events in conflict settings.

The average OPD alert generation time of 7.3 days together with the response time of 2 days or less from alert generation points to the usefulness of the CBS system in both generating an alert and leading to a timely response. This time lag between the alert generation and response time, falls in line with the timeliness observed in the detection, notification and action of alerts between CBS volunteers and supervisors in a study by Byrne and Nichol [13].

The CBS system generated an alert for a suspected case of measles which led to the confirmation of the first ever monkeypox case in the region, with one case previously identified in the Northwest region [14]. This was formally addressed by the Minister of Public Health who called for vigilance from all other health districts in the region. As an OPD, it was added to the list of diseases reportable by the CBS, and telephone training was done on how to identify this disease. This was only possible due to the clear physical signs that this disease presents with. Surveillance was carried out for 21 days, and no further cases were identified.

The collaboration with the district through timely relay of alerts generated by the CBS system allowed the DHS to inform the Regional Delegation of Public Health which alerts the national surveillance system thus leading to an overall increase in the national health surveillance strengthening. The alerts generated by the CBS system led to targeted responses by the MHT which was able to carry out rapid response interventions geared toward reducing suffering, saving lives and containing outbreaks.

The use of CHWs living as part of the displaced communities increased community acceptance and engagement of the communities in mass immunization services, even at short notice. Most of the outbreaks occurred in extremely hard to reach communities where there was little to no government access, including new IDP settlements in bushes. In the absence of an effective CBS system and a ready intervention team, these outbreaks would likely have resulted in higher morbidity and mortality, following the delay witness in organizing a mass measles campaign for the entire district after a confirmed measles outbreak. We believe that the ring vaccination conducted immediately the measles samples were collected, helped in containing the spread of the measles outbreak. This is because after the ring vaccination intervention, no other case of suspected measles was reported, even though a mass measles vaccination only occurred 4 months later. It should be mentioned that the reason behind the delay in having a mass measles campaign in the district was due to delay in receiving vaccines from the central level as the Ministry of Health was planning a national measles campaign and the DHS had to wait for the national campaign dates to be programmed [15].

Communities responded well to feedback on reported outbreaks and this encouraged future reporting, as seen in the review by Guerra [16]. A perceived lack of response could be interpreted as neglect, and lead to reduced future reporting.

Crisis areas are prone to food insecurities which could easily lead to malnutrition, especially amongst under five children [17]. The active screening of children 6 months to 59 months by CHWs and reporting SAM cases through the CBS system led to the early identification and eventual management of 43 children with SAM. CBS of malnutrition prevalence has been used successfully implemented previously [2], however, the use of CHWs trained in treatment of uncomplicated SAM worked well in this setting, even though additional training was required at the start of the project and regular supervision and follow up by the MHT.

Displacement monitoring was carried out as part of the CBS. This was of particular importance in this setting as data about current population figures was lacking and repeated and pendular displacement was common. It is interesting to note the CHWs commonly displaced with the communities so were able to continue surveillance during these pendular displacements. The information on new villages gathered was highlighted to the International Organization of Migration, however, pendular or short-term displacement were commonly not.

Relations between humanitarian actors and the DHS was initially challenging, especially with regards to outbreak reporting. As the traditional reporting system had been predominantly silent in certain health areas, the sudden influx of diseases identified when humanitarian actors began working was viewed negatively. Integration into the existing reporting structure was key for improving these relationships and to avoid duplication. The improved relationship led to over 300 doses of measles and rubella vaccines being provided by the DHS for use by the MHT. This supports *Kuehne* et al*’s* findings that integration is key for rapid verification and response [18].

The extensive acceptance gained through the active participation and collaboration with communities through the service delivery offered as part of the RRM+ model, allowed for community acceptance of the CBS system, and led to safer assess for humanitarian organizations to work, including those coming after the end of the project. Without the other aspects of the project, such as WASH, child protection, health and nutrition, the CBS system would not have been so readily accepted.

After the project ended in April 2020, the MHT informed the district health service of their departure. The district continued working with the CHWs as prior to the start of CBS. However, this time the CHWs had experience from the CBS project which the district will tap from. Further, based on our shared experience, the Ministry of Health in its strategy plan for the conflict affected regions, included scaling up CBS in all districts.

## Conclusion

Implementing community led surveillance of OPDs can help in identifying outbreaks, as has been shown by the confirmation of 3 outbreaks (measles, monkeypox and tropical ulcer), compared to 0 reported by the traditional surveillance system in place in the district in the same time, and 0 in the 3 preceding years. This is especially true in hard-to-reach areas where the tradition system has broken down. The MHT was essential to the success of the CBS, with the ability to constantly respond rapidly to 8 of the 9 alerts generated within an average of 2 days in each case, identifying 3 confirmed outbreaks. It also worked well to identify other diseases or events, such as malnutrition and displacement and successfully identified an outbreak which was previously not included in the CHWs training. Collaboration between the MHT and the DHS worked well, and is possible even in conflict situations. CBS can be successfully added to basic PHC services carried out by CHWs and standardized mobile phone reporting could facilitate alert notification time in areas. Humanitarian actors should explore the use of CBS in humanitarian responses to improve disease surveillance and contribute in strengthening health systems. More research on the cost effectiveness of CBS is also needed in conflict settings and other hard-to-reach humanitarian areas.

## Data Availability

The datasets used during the current project are available from the corresponding author on reasonable request.

## Abbreviations

AFP: Acute flaccid paralysis
ARI: Acute respiratory infection
CBS: Community based surveillance
CHW: Community health workers
CSF: Cerebrospinal fluid
DHS: District Health Service
IDP: Internally displaced person
IOM: International Organization for Migration
MHT: Mobile health team
MUAC: Mid-upper arm circumference
NSAG: Non-state armed group
NWSW: Northwest Southwest
OCHA: Organization for the Coordination of Humanitarian Affairs
OPD: Outbreak prone disease
RDT: Rapid diagnostic tests
RRM +: Rapid Response Mechanism
SAM: Severe acute malnutrition
WHO: World Health Organisation
